# Scalable and safer printed Zn//MnO_2_ planar micro-batteries for smart electronics

**DOI:** 10.1093/nsr/nwz092

**Published:** 2019-07-21

**Authors:** Sang-Young Lee

**Affiliations:** Department of Energy Engineering, Ulsan National Institute of Science and Technology, Korea

The rapidly growing field of miniaturized smart electronics has forced us to search for compatible microscale power sources with reliable electrochemical performance, various form factors, manufacturing scalability, and safety [1–5]. Among the several power sources reported to date, planar micro-batteries, which are characterized by geometrical superiority over simple-stacked ones, have recently garnered considerable attention due to the simple miniaturization, facile serial/parallel integration, mechanical flexibility, and removal of conventional separator membranes [6]. Lithium-based thin-film micro-batteries have been extensively investigated; however, the complex manufacturing processes and flammable organic electrolyteinduced safety concerns pose a formidable barrier to their practical applications. To address this issue, aqueous-based non-lithium planar micro-batteries are suggested as a promising alternative beyond the aforementioned lithium-based ones. They can be fabricated through various printing techniques including inkjet, screen, gravure, and 3D printing [7].

A recent study published in *Natl. Sci. Rev.* by Wu. *et al.* [8] reported a new class of screen-printed, aqueous Zn//MnO_2_ planar micro-batteries as a breakthrough approach. The Zn//MnO_2_ planar micro-batteries, which were based on interdigital patterns of Zn ink as an anode and MnO_2_ ink as a cathode, with high-conducting graphene ink as a metal-free current collector, showed outstanding electrochemical performance, aesthetic diversity, mechanical flexibility, and modularization.

**Figure 1.**
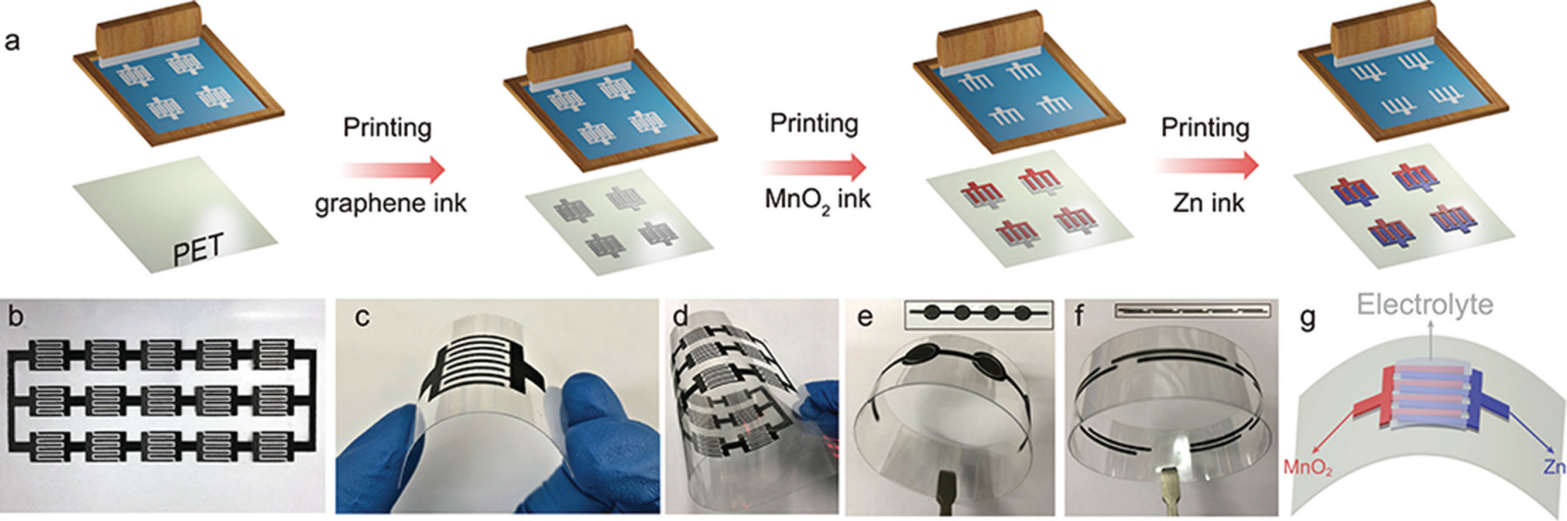
(a) Schematic of fabrication of printed Zn//MnO_2_ micro-batteries. (b) An energy storage pack of Zn//MnO_2_ micro-batteries connected in a tandem fashion of 5 series × 3 parallel. (c–f) Photographs of shape-designable Zn//MnO_2_ micro-batteries under different bending states, e.g. (c) an individual interdigital Zn//MnO_2_ micro-battery, and (d) the tandem energy storage packs via self-connection of (c) interdigital Zn//MnO_2_ micro-batteries in 5 series × 3 parallel bended at 180°, (e) four concentric-circle-shape, and (f) five linear-shape Zn//MnO_2_ micro-batteries in series, under flat and bent (180°) states. (g) Schematic of the bent Zn//MnO_2_ micro-battery with electrolyte.

The Zn//MnO_2_ micro-batteries were fabricated by a low-cost and scalable screen-printing technique as illustrated in Fig. 1a. The screen-printing enabled seamless integration of the Zn//MnO_2_ micro-batteries with various complex-shaped planar geometries, resulting in the fabrication of multiple parallel interdigitated micro-batteries via in-series/in-parallel connections (Fig. 1b), individual micro-batteries (Fig. 1c), flexible patterns with multiple connections (Fig. 1d), and flexible tandem concentric circular (Fig. 1e) and linear-structured micro-batteries free from conventional metal-based interconnectors (Fig. 1f). The planar Zn//MnO_2_ micro-batteries employed neutral aqueous electrolytes (Fig. 1g). They delivered a high volumetric capacity of 19.3 mAh/cm^3^ and, notably, a volumetric energy density of 17.3 mWh/cm^3^, outperforming those (≤10 mWh/cm^3^) of conventional lithium thin-film batteries. The Zn//MnO_2_ micro-batteries also provided long-term cyclability, high capacity retention of 83.9% after 1300 cycles at a current density of 5 C, which far exceeds those of stacked Zn//MnO_2_ batteries reported to date. Furthermore, the Zn//MnO_2_ planar micro-batteries exhibited exceptional flexibility without capacity loss under serious deformation and high voltage/high capacity through facile serial and parallel connection of bipolar cells. The serial or parallel Zn//MnO_2_ planar micro-batteries were assembled with unit cells one by one, which were packaged by dropping electrolyte onto the project area of interdigital microelectrodes.

The low-cost, environmentally benign Zn//MnO_2_ micro-batteries with in-plane geometry presented in this study hold great promise as a high-performance, safe, flexible, and shape-versatile printed microscale power source that can be directly integrated with various miniaturized electronics. This study will be of broad interest to scientists and engineers involved in nanotechnology, chemistry, material science, and energy storage, and contributes to enriching development perspectives and directions of planar microscale power sources for potential use in future microelectronics. Research directions on printable batteries are currently focused on (i) synthesis of highly conducting and stable battery component inks with tunable rheological properties associated with electrochemical performance, (ii) design of battery shapes and configurations with fully printable techniques, (iii) development of industrially scalable printing techniques, and (iv) monolithic/seamless integration of printable batteries with electronic devices [2,9].
